# Patient Acceptable Symptom State in Self-Report Questionnaires and Composite Clinical Disease Index for Assessing Rheumatoid Arthritis Activity: Identification of Cut-Off Points for Routine Care

**DOI:** 10.1155/2015/930756

**Published:** 2015-06-18

**Authors:** Fausto Salaffi, Marina Carotti, Marwin Gutierrez, Marco Di Carlo, Rossella De Angelis

**Affiliations:** ^1^Rheumatology Department, Polytechnic University of the Marche, Jesi, 60035 Ancona, Italy; ^2^Radiology Department, Polytechnic University of the Marche, 60035 Ancona, Italy

## Abstract

*Objective*. To provide information on the value of Patient Acceptable Symptom State (PASS) in rheumatoid arthritis (RA) by the identification of PASS thresholds for patient-reported outcomes (PROs) composite scores. *Methods*. The characteristics of RA patients with affirmative and negative assignment to PASS were compared. Contributors to physician response were estimated by logistic regression models and PASS thresholds by the 75th percentile and receiver-operating characteristic (ROC) curve methods. *Results*. 303 RA patients completed the study. All PROs were different between the PASS (+) and PASS (−) groups (*p* < 0.0001). The thresholds with the 75th percentile approach were 2.0 for the RA Impact of Disease (RAID) score, 2.5 for the PRO-CLinical ARthritis Activity (PRO-CLARA) index, and 1.0 for the Recent-Onset Arthritis Disability (ROAD) questionnaire. The cut-off values for Clinical Disease Activity Index (CDAI) were in the moderate range of disease activity. Assessing the size of the logistic regression coefficients, the strongest predictors of PASS were the disease activity (*p* = 0.0007) and functional state level (0.006). *Conclusion*. PASS thresholds were relatively high and many patients in PASS had moderate disease activity states according to CDAI. Factors such as disease activity and physical function may influence a negative PASS.

## 1. Introduction

Rheumatoid arthritis (RA) is a chronic disabling inflammatory disease with unpredictable course and wide variation in severity, affecting about 0.5% of the population [1]. The evaluation of the disease requires a comprehensive assessment of both inflammatory process and outcome. The lack of a single instrument to assess the global status of the disease leads to adopt multiple measures to determine the different aspects of the underlying disease.

To date, several measures have been proposed for this goal, including joint counts, acute phase reactants, global assessment scales, pain, fatigue, or physical status. However, due to the high variability of the presentation and course of RA, it is a challenge to generate a single measure able to reliably capture the disease activity. Moreover the evaluation of all measures individually is associated with some methodological and statistical problems, especially when they are employed as endpoints in clinical trials. All of these considerations induce a rational for “pooling” the individual measures of disease activity into composite scores.

In order to integrate the different aspects of disease activity and perform the responsiveness, different indexes such as Disease Activity Score, using 28 joint counts (DAS-28) [2], European League Against Rheumatism (EULAR) response criteria (EULARC), as well as Simplified Disease Activity Index (SDAI) [3] or the Clinical Disease Activity Index (CDAI) have been developed [4]. They give the opportunity to perform a “tight control” of the disease which is associated with both significantly better outcomes and high adherence than usual nonquantitative care of RA [5]. In spite of these aspects, it has been recently raised some discussion about the clinical relevance of such indexes with respect to daily practice from the patient's opinion perspective [6, 7].

Data from several clinical trials suggest that a strategic approach to RA treatment, targeting low disease activity, may benefit a large proportion of patients and clearly improve the outcome of the disease [8–10]. In daily life, the decision to change the therapeutic regimen can be made with the patient, but often not including his thoughts and perspectives. Thus in the last few years there is a shift towards a more patient-centered perspective of the disease, adopting the patient-reported outcomes (PROs) [11] which reflect patients' perceptions of their health or treatment, as reported by the patients themselves. The EULAR, American College of Rheumatology (ACR), and Outcomes Measures in Rheumatology (OMERACT) have also well outlined the importance of PROs in addition to physician assessed outcomes for the assessment of both disease progression and responsiveness of RA [12, 13]. Besides, regulatory bodies and healthcare decision entities also recognize the importance of capturing the patient perspective in clinical trials by the PROs [14, 15].

To date, different PROs measure-based composite index such as the RA Disease Activity Index (RADAI) [16], the Patient Activity Scale (PAS) or PAS-II [17], the Routine Assessment of Patient Index Data (RAPID3) [18], the Rheumatoid Arthritis Impact of Disease (RAID) [19], and PRO-CLinical ARthritis Activity (PRO-CLARA) [20] have been proposed. Although they permit the comparison of the disease status of patient groups or individually, there is no consensus on how best to interpret PROs results because a statistically significant change in PROs scores does not necessarily reflect a comparable clinical improvement. In order to interpret better the PROs change in scores in clinical routine, some cut-points have been determined. One of these cut-points is the Patient-Acceptable Symptom State (PASS) that is defined as the highest level of symptom beyond which patients consider themselves well [21, 22]. It is strictly a PRO and consists of a global dichotomized simple question about patient's satisfaction of their state of symptoms.

To provide further information on the value of PASS in daily rheumatology practice we conducted this study aimed to (i) to identify the cut-off points for PASS in CDAI and PRO measure-based composite scores such as RAID and PRO-CLARA and (ii) to determine the clinical variables associated with the PASS.

## 2. Patients and Methods

### 2.1. Study Population

Between March 2012 and May 2014, 303 consecutive RA patients (245 women, 58 men) were enrolled. Eligible patients were >18 years of age, adult-onset RA as defined by the ACR/EULAR classification criteria for RA [23] for at least 3 months. Two hundred fifty-five patients showing an unsatisfactory response or intolerance to at least one conventional disease-modifying antirheumatic drug (cDMARD) (methotrexate, leflunomide, sulfasalazine, or hydroxychloroquine) or at least one biologic DMARD (bDMARD) (infliximab, adalimumab, certolizumab, golimumab, abatacept, or tocilizumab) were included. All patients were classified according to the CDAI as follows: CDAI ≤ 10 = low disease activity; CDAI ≤ 22 = moderate disease activity; CDAI > 22 = high disease activity. A CDAI ≤ 2.8 was considered for remission [24]. All patients gave their informed consent for anonymous analysis of data. This cross-sectional study was approved by our institutional review board of the University Hospital.

### 2.2. Demographics, Disease-Related Characteristics, and Clinical Assessment

A comprehensive questionnaire package, including sociodemographic data, functional measures, and disease-related variables, was administered to the patients. Disease duration (years) was included among disease-related characteristics. Clinical assessment comprised the following single items of disease activity: 28 joint counts for swollen and tender joints (SJC and TJC, resp.) and evaluator and patient's assessments of disease activity (EGA, PTGA, resp.). These variables were used to calculate the composite CDAI [4, 25].

### 2.3. Composite Patient-Reported Disease Activity Scale

PRO-CLARA is a validated, short, and easy to complete self-administered index [20], without formal joint counts, combining three items on patient's physical function (as measured by Recent-Onset Arthritis Disability (ROAD) questionnaire), self-administered TJC and PTGA into a single measure of disease activity [26, 27]. Its total score was completed by summing the scores of the three individual measures and dividing this by three, and range from 0 to 10. The ROAD questionnaire is a reliable, valid, and responsive tool for measuring physical functioning in patients with RA, and it is suitable for use in clinical trials and daily clinical practice [26–28]. The ROAD consists of 12 items assessing a patient's level of functional ability and includes questions related to fine movements of the upper extremity, locomotor activities of the lower extremity, and activities that involve both upper and lower extremities. For each item, patients are asked to rate level of difficulty over the past week on a 5-point scale, which ranges from 0 (without any difficulty) to 4 (unable to do). The ROAD score ranges from 0 to 48. In order to express these scores in a more clinically meaningful format, a simple mathematical normalization procedure was then performed so that all the scores could be expressed in the range 0–10, with 0 representing best status and 10 representing poorest status. The self-administered TJC was evaluated according to joint list of the RADAI [16]. The RADAI joint mannequin list queries pain “today” in 16 joints or joint groups, including left and right shoulders, elbows, wrists, fingers, hips, knees, ankles, and toes. The self-administered TJC weighted the degree of tenderness of each joint on the following scale: 0 = none; 1 = mild; 2 = moderate; 3 = severe. The self-administered TJC is scored as 0–48; the raw 0–48 score may be recoded to 0–10 using the scoring template. The PTGA is scored with the follow question: “Considering all the ways in which illness and health conditions may affect you at this time, please make a mark below to show how you are doing” on a 0–10 numerical rating scale (NRS) with very well (0) and very poorly (10) as anchors.

RAID is a validated composite measure of impact of RA [19] that includes seven domains (pain, function, fatigue, physical and psychological wellbeing, sleep disturbance, and coping). Each domain is evaluated using a single question answered by a 0 to 10 NRS. Each domain also has a specific weight assigned by a patient survey. The RAID score is a continuous variable ranging from 0 (best) to 10 (worst). Finally, patient opinion of their symptoms state (PASS) was recorded as a “yes” or “no” answer to the anchoring question: “Considering all the different ways your disease is affecting you, if you were to stay in this state for the next few months, do you consider that your current state is satisfactory?” [21].

### 2.4. Statistical Analysis

We compared patients in a PASS versus those who reported not being in a PASS, demographic and disease characteristics by descriptive statistics (mean ± SD, median, interquartile range) and using Student's *t*-test for continuous variables or for non-Gaussian variables, Mann-Whitney *U* test. For categorical variables, chi-square test was used. The relationship between PRO measures and CDAI scores was assessed with Spearman's rank-order correlation coefficient. To assess the construct of the PASS concept, the contribution of demographic (age, sex, and disease duration) and clinical variables (CDAI), functional disability (ROAD), and other PRO measures (pain, fatigue, emotional wellbeing, sleep disturbance, or PTGA) to attainment of a PASS condition was analyzed by stepwise logistic regression. We further examined the construct validity of the PASS by comparing proportions of PASS positive and PASS negative patients who met the CDAI criteria for remission. Finally, the threshold at which patients considered themselves in PASS for each of the PRO measures and clinical composite score was estimated using three different approaches: (i) as the 75th percentile of the cumulative distribution for each outcome for patients who rated their condition as PASS positive; (ii) by plotting receiver-operating characteristics (ROC) curves and identifying cut-offs that yielded 80% specificity; (iii) by plotting ROC curves and identifying cut-offs that yielded the smallest number of false-positives and false-negatives [21, 22]. The external anchor was the general question on PASS. Results are expressed as OR (95% CI). All analyses were performed with SPSS software (Windows release 11.0; SPSS Inc., Chicago, Illinois, USA), and MedCalc, version 10.5.1 for Windows XP.

## 3. Results

### 3.1. Description of PASS

The characteristics of the entire PASS and PASS positive and PASS negative patients are separately shown in Table 1. Fifty-five RA patients out of 303 (18.2%) resulted in PASS. Patients in PASS reported less total pain, less stiffness, and fatigue, lower CDAI scores, better function (ROAD), better physical and emotional wellbeing, and coping (*p* < 0.0001 for all comparisons with PASS negative patients). Patients in PASS were also significantly older (59.7 ± 9.6 versus 54.1 ± 10.1; *p* = 0.041) and had a somewhat longer disease duration (6.3 ± 4.1 versus 4.9 ± 3.7; *p* = 0.037).

### 3.2. Thresholds of PROs for Being in PASS

PASS thresholds for the RA self-PROs (RAID and PRO-CLARA) and for composite CDAI (as defined by the 75th percentile of the cumulative distribution for each specific outcome) and the percentages of patients with scores below these thresholds but still reporting not being in PASS are listed in Table 2. Patients who considered their state satisfactory rated their RAID and PRO-CLARA score of 2.0 and 2.5, respectively, on the 0–10 scale. Similarly, patients who considered their state satisfactory rated their functional status (ROAD) and patient' global assessment score of 1.0 and 3.3, respectively, on the 0–10 scale. PASS cut-off determined by ROC analysis demonstrated somewhat higher values. The threshold for CDAI using the 75th percentile was 13.8 and using the ROC curve approach was 15.8 (80% specificity cut-off) and 14.2 (ROC cut-off). According to the defined CDAI cut-off values for disease activity states, most of values corresponded to moderate disease activity. In fact only 15 (27.3%) RA patients in PASS out of 55 were considered in remission (CDAI ≤ 2.8), whereas 18 (32.7%) and 22 (40%) RA patients in PASS demonstrated low disease activity (CDAI > 2.8 ≤ 10) and moderate disease activity (CDAI ≤ 22), respectively.

### 3.3. Variables Associated with PASS

In the logistic regression model (Table 3), lower CDAI (OR 0.71, 95% CI 0.58–0.86; *p* = 0.0006) and lower physical disability score in ROAD (OR 0.23, 95% CI 0.07–0.75; *p* = 0.0151) were independent variables associated with being in PASS. Other predictors such as age, sex, disease duration, and emotional wellbeing were not clinically important contributors to PASS. Area under the ROC curve (AUC) of the regression model was 0.95 (95% CI 0.92–0.97).

## 4. Discussion

Recent studies have emphasized a discrepancy between patients and physicians rates for disease activity of RA [29, 30]. It is clear that this discordance can negatively affect the patient care, treatment compliance and adherence, disease outcome, and productivity with consequent increase of cost to society [31]. Clearly, physicians are most focused on “RA-specific outcomes,” whereas patients are more focused on how the general health state is affected by RA [32]. The PASS is a new PRO concept that reflects the overall health state at which patients consider themselves well, an absolute level of wellbeing. It consists of a global dichotomized simple question about patients satisfaction with their state of symptoms [21, 22]. The identified cut-off point for PASS may easily be incorporated as endpoints in clinical trials and will provide information about the proportion of patients achieving acceptable state of symptoms. The concept of PASS is widely supported by the OMERACT international network, which has focused various meeting sessions on this issue [33] and has contributed importantly to understanding in this area.

An important body of evidence supporting the utility of PASS is currently available. It has been employed in patients with osteoarthritis [34], acute painful shoulder [35], low back pain [34], systemic lupus erythematous [36], ankylosing spondylitis (AS), and RA and psoriatic arthritis (PsA) [21, 22, 34, 37]. The results have demonstrated a significant association between PASS and disease activity or severity evaluated with different indexes [34, 35]. In patients affected by AS, a significant association between the presence of an acceptable symptom state and a reduced disease activity, assessed with Bath Ankylosing Spondylitis Disease Activity Index (BASDAI), Bath Ankylosing Spondylitis Functional Index (BASFI), and/or Ankylosing Spondylitis Disease Activity Score (ASDAS) cut-off, was found, underlining the external validity of PASS [38–41]. More recently, PASS was administered to patients affected by RA. It has been shown that a positive response to PASS, when using the external anchoring question focusing on “satisfactory condition,” is associated with a range of moderate disease activity, assessed with several composite indices, such as DAS28, CDAI, and SDAI [42]. This observation agrees with our results. For the estimation of PASS cut-off points, we used the validated 75th centile method as the primary tool [43], but we also performed ROC analyses to study the robustness of the cut-off points across approaches. The PASS cut-off points of disease activity levels identified in our study exceeded the remission and low disease activity state. Forty percent of patients in PASS had a moderate activity state according to the proposed cut-off values of CDAI [25]. Moreover PASS threshold of CDAI which determined either the 75th percentile or ROC analysis resulted also in a range of moderate disease activity. However it is important to underline that the CDAI scores in these patients were closer to the lower threshold than high.

The identified PRO-CLARA values corresponding to PASS was 2.5 with the 75th centile approach and 2.9 in the ROC analyses [20, 44]. For the ROAD the identified PASS cut-off point value was 1.0 with 75th centile, which is similar to the average HAQ score found in large patients populations. The RAID cut-off points for PASS condition in our cohort of patients with RA were close to others [45]. In particular, Dougados et al. found that a change of at least 3 points (absolute) or 50% (relative) in the RAID score should be used to define a minimal clinically important improvement and that a maximal value of 2.0 defines an acceptable status [46].

The variability in PROs thresholds defining the PASS is potentially problematic with respect to its use in clinical practice and research and suggests the influence of important confounders that are unrelated to treatment and could affect the attainment of PASS. This variability may originate from several sources, including patients' experiences of disease, dynamics of the patient and physician relationship, psychological factors, cultural factors, random factors, and other systematic differences between patients and physicians' assessments of disease activity or severity.

In patient with SA, Maksymowych et al. [21] demonstrated that PASS condition was independently associated with increasing age, lower patient assessment of global disease activity, and better functional status (BASFI). In contrast, in the placebo-controlled clinical trial of adalimumab, age < 40 years was independently associated with attainment of the PASS [38]. Rodríguez-Lozano et al. [40] found that lower BASDAI and lower physician global assessment were independent variables associated with being in PASS, whereas other clinical or demographic variables such as age, sex, disease duration, occupation, or education level were not associated with PASS status. Similar results were reported in a study of PASS in hip and knee osteoarthritis [43].

In our logistic model we found that lower CDAI and lower physical disability score were independent variables associated with being in PASS. Age, sex, disease duration, and emotional wellbeing were no contributors to PASS. Previously, we demonstrated that the physical component of the 36-Item Short Form Health Survey (*SF*-*36*) was influenced (at a *p* level < 0.0001) by a high disease activity (measured by DAS28) in RA [45]. A similar association was found in patients with AS, peripheral PsA, and axial PsA [45]. Kirwan, [47] similarly, concluded that disease activity remains the major determinant of disability in RA, both late in disease and in patients with substantial radiographic damage.

Our study has some limitations. The cross-sectional design allowed assessment of patient's situation in only a single visit. Patients' perceptions of disease vary according to whether there has been an improvement or worsening of health compared to the past [48]. The perception of improvement of disease activity of patients with RA is considerably different depending on the disease activity level at which they start [49]. Another potential weakness is that the proposed thresholds were defined in a single centre within a relatively small region and that the study population in general, although being representative of the centre's entire RA patient population, was mildly to moderately diseased. On the other hand, the strength of this study is that all these different analyses of the definition of thresholds for a continuous variable were performed on a uniform group of patients. Finally, the measures to assess the patients' perspective, namely, RAID and PRO-CLARA, have not yet been validated completely. For further validation, we would propose to include the patients' perspective, particularly the patients' therapeutic attitude, into clinical trials as well as into multicentre observational investigations.

In conclusion, this study showed that the identified PASS thresholds for common recommended outcome measures were relatively high and many patients in PASS had a moderate disease activity state, according to CDAI. In the light of this, PASS accentuates the importance of taking PROs measures into consideration in treatment decisions, but it should not be used as the ultimate therapeutic goal such as low disease activity or remission [5, 6]. Longitudinal studies are needed to draw definitive conclusions on the value of PASS as an outcome measure in RA.

## Figures and Tables

**Table 1:** Demographic and disease severity characteristics of patients with RA according to PASS status.

	PASS negative				PASS positive				All patients			
	Answer, *n* = 248				Answer, *n* = 55				*N* = 303			
	Mean	SD	Median	IQR	Mean	SD	Median	IQR	Mean	SD	Median	IQR
Women sex, number (%)	201 (82.0)				44 (80.0)				245 (80.8)			
Age, mean ± SD years	54.1 ± 10.1				59.7 ± 9.6				56.9 ± 9.9			
Disease duration, mean ± SD years	4.9 ± 3.7				6.9 ± 4.1				5.9 ± 3.9			
PRO-CLARA (0–10 scale)	4.026	1.2748	3.920	3.020–4.715	1.969	0.8057	1.960	1.422–2.510	3.652	1.4407	3.540	2.755–4.578
(i) ROAD (0–10 scale)	2.679	2.0410	2.500	0.630–3.960	0.861	0.5924	0.630	0.420–1.040	2.349	1.9906	2.080	0.630–3.130
(ii) Self-administered TJC (0–10)	1.609	1.5868	1.040	0.525–2.080	0.721	0.4474	0.630	0.420–0.830	1.448	1.4875	0.830	0.420–1.880
(iii) Patient's assessment of disease activity (0–10 NRS)	7.790	1.7554	8.000	7.000–9.000	4.618	2.4381	5.000	3.000–6.000	7.215	2.2545	8.000	6.000–9.000
RAID (0–10 scale)	4.554	2.1946	4.935	2.405–6.070	1.709	0.9731	1.760	1.145–2.098	4.038	2.3055	3.760	1.897–5.790
(i) Pain (0–10 NRS)	3.573	2.5600	3.000	1.500–5.000	1.745	1.5542	1.000	1.000–2.000	3.241	2.5079	3.000	1.000–5.000
(ii) Functional disability (0–10 NRS)	4.690	2.4978	5.000	2.000–7.000	2.018	1.2396	2.000	1.000–2.000	4.205	2.5380	4.000	2.000–6.000
(iii) Fatigue (0–10 NRS)	5.153	3.0977	5.000	2.000–8.000	2.382	1.8806	2.000	1.000–3.000	4.650	3.1025	4.000	2.000–8.000
(iv) Sleep (0–10 NRS)	2.431	2.7076	2.000	0.000–4.000	0.582	1.1497	0.000	0.000–1.000	2.096	2.5966	1.000	0.000–3.000
(v) Physical wellbeing (0–10 NRS)	2.919	2.5343	2.000	1.000–4.000	1.436	1.2136	1.000	1.000–2.000	2.650	2.4175	2.000	1.000–4.000
(vi) Emotional wellbeing (0–10 NRS)	4.577	3.3407	5.000	1.000–8.000	1.327	1.8059	1.000	0.000–2.000	3.987	3.3593	3.000	1.000–8.000
(vii) Coping (0–10 NRS)	7.698	1.9198	8.000	6.000–9.000	3.891	2.8328	4.000	1.000–6.000	7.007	2.5708	8.000	6.000–9.000
CDAI (0–76)	20.538	5.5005	19.830	17.310–24.460	8.757	6.1678	8.210	2.79–13.83	18.407	7.2233	18.710	15.080–23.420

IQR = interquartile range (25th–75th percentile); PRO-CLARA = patient-reported outcome-CLinical ARthritis Activity; ROAD = Recent-Onset Arthritis Disability; TJC = tender joint count; RAID = Rheumatoid Arthritis Impact of Disease; CDAI = Clinical Disease Activity Index.

**Table 2:** PASS thresholds for each measure defined by the 75th percentile of the cumulative distribution, for patient who rated their condition as PASS positive, by plotting ROC curves and identifying cut-offs that yielded 80% specificity and by plotting ROC curves and identifying cut-offs that yielded the smallest number of false-positives and false-negatives.

Variable	PASS + 75th percentile threshold	80% specificity cut-off	ROC cut-off	Sensitivity/specificity	AUC-ROC curve
ROAD (0–76)	1.00	1.10	1.50	92.7/60.1	0.788
PRO-CLARA (0–10)	2.50	2.60	2.90	89.1/82.7	0.933
RAID (0–10)	2.00	2.10	2.60	96.4/73.4	0.862
CDAI (0–76)	13.83	15.85	14.21	81.8/91.6	0.925

**Table 3:** Logistic regression analysis: coefficients and standard errors, odds ratios, and 95% confidence intervals.

Variable	Coefficient	Std. error	Odds ratio	95% CI	*p*
CDAI (0–10)	−0.33992	0.09928	0.7118	0.5859 to 0.8648	0.0006
ROAD (0–10)	−1.46604	0.60355	0.2308	0.0707 to 0.7535	0.0151
Emotional wellbeing (0–10 NRS)	−0.19771	0.16329	0.8206	0.5959 to 1.1301	0.2260
Pain (0–10 NRS)	0.53754	0.26423	1.5118	1.0198 to 2.8732	0.0619
Fatigue (0–10 NRS)	0.18332	0.20360	1.2012	0.8059 to 1.7903	0.3679
Sleep (0–10 NRS)	−0.35467	0.31685	0.7014	0.3769 to 1.3052	0.2630
Patients assessment of general health (0–10 NRS)	−0.08423	0.20027	0.9192	0.6208 to 1.3611	0.6740
Age (years)	−0.48198	0.57029	0.6176	0.2019 to 1.8885	0.3980
Sex	0.30136	0.42691	1.3517	0.5854 to 3.1208	0.4803
Disease duration (years)	0.84826	0.43536	2.0356	0.9950 to 5.4825	0.0714
*Constant *	*4.2761 *				
